# Chemokine (C-C motif) Ligand 2 is a potential biomarker of inflammation & physical fitness in obese children: a cross-sectional study

**DOI:** 10.1186/1471-2431-13-47

**Published:** 2013-04-04

**Authors:** M Constantine Samaan, Joyce Obeid, Thanh Nguyen, Lehana Thabane, Brian W Timmons

**Affiliations:** 1 Department of Pediatrics, McMaster University, Hamilton, Ontario, Canada; 2 Division of Pediatric Endocrinology, Department of Pediatrics, McMaster Children’s Hospital, McMaster University, 1280 Main Street West, HSC-3A57, Hamilton, Ontario, L8S 4K1, Canada; 3 Population Health Research Institute, Hamilton, Ontario, Canada; 4 Department of Clinical Epidemiology and Biostatistics, McMaster University, Hamilton, Ontario, Canada; 5 Department of Anesthesia, McMaster University, Hamilton, ON, Canada; 6 Centre for Evaluation of Medicines, Hamilton, ON, Canada

**Keywords:** Childhood obesity, Immunometabolism, Inflammation, Chemokine C-C Ligand 2, Fitness

## Abstract

**Background:**

Obesity is a global epidemic that is impacting children around the world. Obesity is a chronic inflammatory state with enhanced production of multiple cytokines and chemokines. Chemokine (C-C motif) Ligand 2 (CCL2) is produced by immune and metabolic cells and attracts immune cells into liver, muscle and adipose tissue, resulting in initiation and propagation of the inflammatory response in obesity. How obesity and fitness affect the production of this chemokine in children is unknown.

This study tested the hypotheses that CCL2 levels are higher in obese children when compared to lean controls, and that fitness modulates CCL2 levels allowing its use as a biomarker of fitness.

**Methods:**

This was a cross sectional case–control study conducted in a Pediatric Tertiary care center in Hamilton, Ontario, Canada. Controls were recruited from the community. This study recruited overweight/obese children (BMI ≥ 85^th^ percentile, n = 18, 9 female, mean age 14.0 ± 2.6 years) and lean controls (BMI < 85^th^ percentile, n = 18, 8 female, mean age 14.0 ± 2.6 years) matched for age, sex and biological maturation.

Aerobic fitness test was done using a cycle ergometer performing the McMaster All-Out Progressive Continuous Cycling test to exhaustion to determine peak oxygen uptake. Fasting CCL2 samples were taken prior to test. Categorical variables including subject categorization into different aerobic fitness levels in overweight/obese and lean children was reported based on the median split in each group.

**Results:**

Obese participants had significantly higher CCL2 levels when compared to lean group (150.4 ± 61.85 pg/ml versus 112.7 ± 38 pg/ml, p-value 0.034).

To establish if CCL2 is a biomarker of fitness, we divided the groups based on their fitness levels. There was a main effect for group (F (3,32) = 3.2, p = 0.036). Obese high fitness group were similar to lean unfit and fit participants. Post-hoc analysis revealed that the overweight/obese low fitness group had significantly higher level of CCL2 compared to the lean low fitness group when adjusted to age, sex and maturity offset (F (3,29) = 3.1, p = 0.04).

**Conclusions:**

CCL2 serves a dual role as a potential biomarker of inflammation and fitness in obese children.

## Background

Obesity is a protective evolutionary mechanism that helped humankind survive famine and, as such, is as old as humanity. This once adaptive mechanism has become counterproductive in modern society, due to unprecedented lifestyle changes that have resulted in an obesity epidemic on a global scale, with 1.5 billion adults and 200 million children and adolescents being overweight or obese [1-4].

Some of the obesogenic factors in the environment include the constant availability and affordability of food in general, and especially processed foods and sugary drinks [5-7]. In addition, the reduction in physical activity at home and in school [8], the reliance on the use of technology, and shorter duration of sleep [9] are some of the precursors involved in the genesis of childhood obesity. These elements interact with each other and with genetic and epigenetic factors to mediate the body’s response to excess weight, and this is an area of intensive research. However, the mechanisms that lead to the initiation of obesity are not yet identified, and understanding the mechanisms that start and propagate obesity will help define interventions for its treatment and prevention.

The significance of childhood obesity lies in its association with other comorbidities in children including glucose intolerance, type 2 diabetes, dyslipidemia, hypertension, obstructive sleep apnea, gastroesophageal reflux, and joint problems [10]. In addition, many obese children are likely to become obese adults, with increased risk of adverse health outcomes including cardiovascular disease and diabetes [10,11].

Pediatric weight management programs focus on life style interventions to manage obesity, and one pillar of these programs is exercise [12]. An important aspect of designing exercise regimens involves the determination of fitness levels to aid with the provision of a targeted exercise plan. Fitness testing requires the existence of a significant infrastructure of equipment and trained personnel, adding to the complexity and cost of care provided. To date, there has been no fitness biomarkers identified that allows us to allocate children to specific exercise programs without the need for costly exercise testing.

On a mechanistic level, obesity is associated with a chronic low-grade inflammatory state [13], characterized by activation of the innate immune system and infiltration of immune cells into metabolic organs including adipose tissue, skeletal muscle and liver [14,15].

The signals that attract immune cells into these organs include a set of cytokines, called chemokines, that have the ability to regulate leukocyte traffic into tissues [16].

One such chemokine is Chemokine (C-C motif) Ligand 2 (CCL2), also known as Monocyte Chemoattractant Protein-1 (MCP-1). The adipocyte is a major source of CCL2 in obesity, the production of which is triggered when cells are exposed to inflammatory cytokines and fatty acids [17], and recently, microRNAs 126 and 193b have also been implicated in the regulation of CCL2 secretion in obese adipose tissue [18]. Other cells are capable of secreting CCL2 in obesity including hepatocytes [19], skeletal muscle cells [15], monocytes, vascular smooth muscle and endothelial cells [20].

The role of CCL2 in childhood obesity is not well studied. Circulating CCL2 levels are increased in obese adults and children [21,22], and correlate positively with BMI and other inflammatory markers like C-Reactive Protein and Interleukin-6 and negatively with High Density lipoprotein (HDL) [23]. CCL2 administration causes insulin resistance in mice [24], although there is conflicting evidence to its correlation with insulin resistance in adult humans [23,25,26].

In addition, CCL2 has also been implicated in monocyte infiltration into atherosclerotic plaques and exacerbation of atherosclerosis [27]. Weight loss and exercise lead to a reduction in CCL2 levels, and improved insulin sensitivity [22,28].

While CCL2 has been implicated in inflammation and insulin resistance, its correlation with fitness has not been studied previously. As CCL2 levels are elevated in obesity and is associated with inflammation and insulin resistance, and as exercise lowers CCL2 levels, the aim of this study was to test the hypothesis that CCL2 levels are higher in obese children when compared to lean controls, and that CCL2 levels are higher in children with low fitness levels when compared to fit children reflecting a more robust inflammatory response. We predicted that this molecule could serve a dual role as a biomarker of fitness and inflammation in children.

## Methods

### Study design and protocol

This was a cross sectional case – control study conducted at a tertiary pediatric center in Hamilton, Ontario, Canada. The Hamilton Health Sciences/Faculty of Health Sciences Research Ethics Board approved the study protocol, and the study was conducted in compliance with the declaration of Helsinki for protection of human research subjects. The primary outcome of the study is CCL2 differences between lean and overweight/obese participants, and the secondary outcome is the detection of differences between lean and overweight/obese groups based on fitness levels.

### Study participants

A total of 18 overweight or obese children (7 with BMI between 85^th^–95^th^ percentile, 11 with BMI ≥95^th^ percentile, 9 female, mean age 14.0 ± 2.60 years) and 18 lean controls (BMI < 85^th^ percentile, 8 female, mean age 14.0 ± 2.60 years) were matched for chronological age, sex and biological age, where pairs were ≤1 year of estimated years from the age of peak height velocity [29]. Overweight and obese children were recruited from the weight management program at the Children’s Exercise & Nutrition Centre and lean controls were recruited from the local community through advertisements distributed through schools. Participant characteristics are shown in Table 1.

**Table 1:** Characteristics of study participants

** Variable**		**Lean (N = 18)**	**Overweight/obese (N = 18)**	**P-value**
**Sex**	**F:M**	8:10	9:9	1.00
**Age (years)**	**Mean**	14.00	14.00	0.99
	**SD**	2.60	2.60	
**YPHV (years)**	**Mean**	1.10	1.50	0.47
	**SD**	2.10	2.10	
**Height (m)**	**Mean**	1.65	1.66	0.99
	**SD**	0.16	0.16	
**Weight (kg)**	**Mean**	52.60	82.40	<0.001
	**SD**	12.90	28.80	
**BMI (kg/m**^**2**^**)**	**Mean**	19.00	29.3	<0.001
	**SD**	2.20	6.80	
**BMI percentile**	**Mean**	46.20	95.00	<0.001
	**SD**	26.30	4.90	
**%FM DEXA**	**Mean**	18.00	33.80	<0.001
	**SD**	6.60	9.20	
**VO**_**2**_**max (ml/kg/min)**	**Mean**	53.50	38.60	<0.001
	**SD**	6.80	11.10	
**VO**_**2**_**max-Lean (ml/kg Lean/min)**	**Mean**	66.40	58.60	0.016
	**SD**	8.60	9.80	

YPHV = years from peak height velocity; BMI = body mass index;%FM DEXA = percentage of fat mass using dual x-ray absorptiometry; VO_2_max = maximal oxygen consumption; VO_2_max-Lean = maximal oxygen consumption adjusted to lean body mass; SD = standard deviation. Independent sample t-test was used for analysis.

### Protocol

Upon full explanation of the study protocol, participants signed an assent form and parents/guardians signed the consent form.

Participants’ height (Harpenden Stadiometer) and weight (BWB-800, Tanita Corporation, Japan) were then measured, along with body composition as assessed by dual energy x-ray absorptiometry (DEXA), and lung function using standard spirometry tests. An aerobic fitness test performed on a cycle ergometer was then used to determine peak oxygen uptake as per the previously described McMaster All-Out Progressive Continuous Cycling test [30]. Progression in this test is achieved by an increase in resistance every 2 min. The test is constructed according to body height such that the total exercise time will range from 8 to 12 min for most children. In adults, it is common to assign specific criteria to determine whether a maximal test has been achieved. In children, these criteria have little value; for example, a plateau in VO_2_ with increasing power output occurs in only about 50% of children, and equations of age-predicted maximum heart rate are not accurate during childhood because maximal heart rate remains constant during growth. Therefore, the pediatric-specific criteria we used included: a heart rate of ≥195 beats per minute, a respiratory exchange ratio (RER) ≥ 1.0, and an inability to maintain the prescribed pedaling cadence in spite of strong verbal encouragement.

In our experience, a motivated child who is attempting to maintain the appropriate cadence, but cannot do so, in spite of encouragement is very likely to have reached their limit. Measurements of expired O_2_ and CO_2_ were made continuously using a calibrated metabolic cart (VMAX 29, SensorMedics, Yorba Linda, CA, U.S.A.), with appropriately sized pediatric mouthpieces.

### Blood sampling & analysis

Serum samples were collected following a 10-hour overnight fast. Fasting lipids (triglycerides, cholesterol, LDL, and HDL) were analyzed at the McMaster University Medical Center core laboratory using Roche P Module and standard Roche reagents. Serum samples were analyzed for fasting glucose using an assay kit (Cayman Chemical Company, Ann Arbor, Michigan), fasting insulin using an ELISA kit (Invitrogen Corporation, Carlsbad, California), and CCL2 using R&D systems CCL2/MCP-1 Quantikine human ELISA kit (Cedarlane, Oakville, Ontario). The intra-assay variation coefficients were 2.8%, 10.4% and 6.2%, respectively.

### Statistical analysis

Statistical analysis was done using IBM SPSS Statistical Package Version 20 (IBM SPSS Statistics for MAC, Version 20.0. Armonk, NY: IBM Corp) and STATISTICA 5.0. (StatSoft, Tulsa, OK) for fitness/fat mass comparisons. The data were checked for completeness and determined to be free of errors prior to statistical analysis.

Categorical variables including subject categorizations into different aerobic fitness levels in overweight/obese and lean children are reported based on the median split in each group. Continuous variables are reported as mean ± standard deviation (SD). For subgroup analysis, the participants were divided based on their fitness levels to lean-low fitness (L-LF), lean high-fitness (L-HF), overweight/obese low-fitness (OW-LF) and overweight /obese high-fitness (OW-HF) groups.

To calculate study power based on the number of paired participants, we used SP software (version 3.0.43) [31]. Based on previous evidence, and using a mean CCL2 of 222 pg/ml for obese children and 184 pg/ml for lean children, we have power of 1.0 for detecting differences between the mean CCL2 in obese compared to lean children [22,31].

We tested cases and controls for differences between the various variables, with comparisons between groups were conducted using independent sample t-test. CCL2 and HOMA-IR were log transformed to ensure normalization of data. Correlations were tested to explore the relation of fitness and CCL2 levels using CCL2 and fitness adjusted to lean body mass (VO_2_max-Lean) as the dependent variables, and covariates including anthropometric measures, body mass index (BMI), maturity offset, glucose, insulin, Homeostasis Model of Assessment-Insulin Resistance (HOMA-IR), and lipid levels. Fitness and CCL2 subgroup correlations were done using analysis of covariance (ANCOVA) with CCL2 as the dependant variable and fitness level as the independent variable, adjusting for chronological age, sex and maturity offset. The criterion for statistical significance was set at alpha = 0.05, adjusted for multiple analysis using the Bonferroni method.

## Results

The clinical, anthropometric and fitness measures are detailed in Table 1. The groups were matched for sex, chronological age, and maturity offset. The levels of fasting glucose, fasting insulin, HOMA-IR, and fasting lipids were similar in the two groups as noted in Table 2.

**Table 2:** Metabolic parameters in study participants

**Variable**	**Lean**		**Overweight/Obese**		**Difference (95% CI)**	**P-value**
	**Mean**	**SD**	**Mean**	**SD**		
**Insulin (μIU/ml)**	17.50	18.30	14.60	13.20	2.00 (−8.70, 12.70)	0.71
**FBG (mmol/l)**	4.70	0.55	4.80	0.80	0.10 (−0.43, 0.64)	0.70
**HOMA-IR**	2.81	3.71	2.81	2.86	0.04 (−2.40, 2.40)	0.97
**Cholesterol (mmol/l)**	3.90	0.90	4.10	0.70	−0.25 (−0.81, 0.31)	0.38
**Triglycerides (mmol/l)**	0.70	0.30	0.90	0.40	−0.18 (−0.44, 0.07)	0.16
**HDL (mmol/l)**	0.99	0.24	0.88	0.20	0.05 (−0.11, 0.21)	0.55
**LDL (mmol/l)**	2.60	0.90	2.80	0.60	−0.21 (−0.72, 0.30)	0.41
**TC/HDL**	4.10	1.40	4.80	1.20	−0.41 (−1.30, 0.51)	0.37

FBG = fasting blood glucose; HOMA-IR = Homeostasis Model of Assessment-Insulin Resistance; HDL-C = high-density lipoprotein cholesterol; LDL-C = low-density lipoprotein cholesterol; TC/HDL = total cholesterol/ high-density lipoprotein ratio; SD = standard deviation. Independent sample t-test was used for analysis.

**Figure 1:**
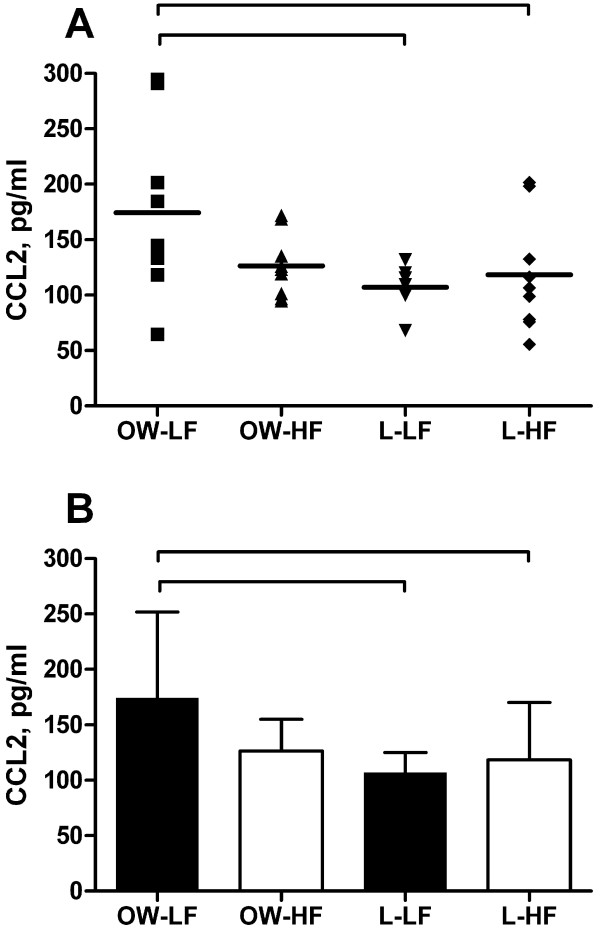
**CCL2 in lean and overweight/obese children according to aerobic fitness.** Study participants (n = 36) were age, sex and maturity offset matched to lean controls. Fasting blood samples were analyzed for CCL2 (intra-assay CV = 6.2%). Aerobic fitness was measured using a continuous cycling test to exhaustion, with values expressed relative to fat-free mass. Group assignments were based on median split of aerobic fitness values in overweight/obese and lean children: overweight/obese, low fitness (OW-LF); overweight/obese, high fitness (OW-HF); lean, low fitness (L-LF); lean, high fitness (L-HF). **A**) Individual values of CCL2 **B**) Combined group values. Significant correlations (P-value <0.05) are marked for comparisons between obese children who have low fitness (OW-LF) and lean children who have low (L-LF) and high (L-HF) fitness levels.

To determine the effect of obesity in children on CCL2 levels, the lean and obese CCL2 groups were compared, and there was a statistically significant difference in CCL2 levels between lean and obese children when analyzed as a group (150.4 ± 61.85 pg/ml versus 112.7 ± 38 pg/ml) with a main effect for the OW-LF group (F (3,32) = 3.2, p = 0.034) when compared to L-LF group (Figure 1). In order to explore what factors contribute to the differences between the two groups, we used spearman’s correlation and multiple regression analyses with CCL2 and variables including VO_2_max adjusted to lean body mass (VO_2_max-Lean), sex, age, maturity offset, BMI, DEXA-measured fat mass percentage, HOMA-IR, cholesterol, triglycerides, HDL, LDL, and total cholesterol/HDL ratio. The result showed that on Spearman’s correlation, BMI percentile and fat mass correlated positively with CCL2 levels (Table 3), and multiple regression analysis showed that BMI is associated with CCL2 levels (β12.43, SE 4.93, 95% CI (1.84, 23), p =0.02).

**Table 3:** Spearman’s correlation analysis of CCL2 and variables including clinical, biochemical and metabolic factors

**Variable**	**Spearman’s correlation coefficient**	**P-value**
**Age/years**	−0.178	0.285
**Sex**	0.062	0.709
**YPHV**	−0.212	0.201
**BMI Percentile**	0.414	0.010
**LOG HOMA-IR**	−0.038	0.836
**%FM DEXA**	0.328	0.048
**VO2max-Lean**	−0.241	0.151
**Cholesterol**	0.222	0.239
**TG**	0.133	0.483
**HDL**	−0.100	0.601
**LDL**	0.193	0.306
**TC/HDL**	0.241	0.200

Correlation coefficients with P-values and 95% confidence intervals are displayed.

Log HOMA-IR = logarithmic transformation of HOMA-IR;%FM DEXA = percentage of fat mass on DEXA scans; VO2MAX Lean = VO2 MAX adjusted to lean mass; TG = Triglycerides; HDL = high-density lipoprotein; LDL = low-density lipoprotein; TC/HDL = total cholesterol to HDL ratio.

To establish if CCL2 is a biomarker of fitness, we divided the groups based on their fitness levels, and then we compared CCL2 levels in L-LF, L-HF, OW-LF and OW-HF groups. There was a main effect for group (F (3,32) = 3.2, p = 0.036). Post-hoc analysis revealed that the OW-LF group had higher level of CCL2 compared to the L-LF group (p-value 0.034). When adjusted to age, sex and maturity offset, the main effect for group remained (F (3,29) = 3.1, p = 0.04). Post-hoc analysis revealed that the OW-LF group had higher CCL2 levels compared to both the L-LF (P-value 0.009) and the L-HF group (p = 0.035). Importantly, OW-HF children had CCL2 levels similar to L-LF and L-HF groups (Figure 1).

## Discussion

In this study, we demonstrate that overweight and obese children have higher circulating CCL2 levels when compared to lean children.

OW-LF children have higher CCL2 concentrations compared to L-LF and L-HF children adjusted for chronological age, sex and maturity offset. Another important observation is that OW-HF children have similar CCL2 levels to lean children. Thus, fitness and fatness modulate CCL2 levels in children.

Our results are in agreement with another recent study that showed CCL2 levels were elevated in obese compared to lean children, and in that study CCL2 levels correlated with insulin resistance. CCL2 levels were reduced when substantial weight loss was achieved, probably indicating an improvement of fitness, and its levels increased in those who did not lose weight [22]. The differences between that study and ours is that our lean and obese participants had similar insulin sensitivity, while Roth *et al.* study participants had significant differences in their insulin levels and HOMA-IR based on their BMI. This is a strength in our study, as it takes out insulin resistance as a factor associated with obesity-related inflammation, and isolates the effect of obesity on CCL2 levels. In addition, lipid levels that may have explained the differences in insulin sensitivity were not reported in the study by Roth *et al*. Our lean and obese participants had similar lipid levels, which may be the result of less lipolysis with equivalent insulin sensitivity in the obese group. In addition, the standardization of fitness assessment using a formal exercise test, and the fact that participants were matched for age, sex and biological maturity is a unique component of our study.

Therefore, we propose that CCL2 serves a dual role as a potential biomarker of fitness and inflammation in obese children; CCL2 may aid the definition of fitness levels and demarcate a group of children who have more inflammation despite having similar metabolic profiles to other obese children but may require more intensive interventions to treat obesity-related inflammation.

A limitation of our study is the inability to determine the source of CCL2; while the main source is probably the adipocyte, fat mass did not differ between the OW-LF and OW-HF groups. Possible explanations for this observation are that the OW-LF group has larger and more inflammatory adipocytes that produce more CCL2, or that CCL2 is secreted by other tissues that were not the subject of our investigation, like skeletal muscle, liver, endothelium or monocytes. In obese adults, circulating CCL2 and gene expression levels are higher in visceral compared to subcutaneous adipose tissue, and this correlates directly with adipose tissue macrophage content and larger adipocytes. This process is not studied in children in visceral fat but in subcutaneous adipose tissue it appears to be less robust than in adults, although macrophage content does correlate with adipocyte size and both have an impact on the inflammatory response in obesity [32]. Whether the OW-LF group has larger adipocytes and more macrophage infiltration into the adipose tissue that correlates with their higher CCL2 levels is unknown, but this group has a more robust inflammatory response based on their CCL2 levels, indicating that inflammation is an important factor in their obesity.

While levels of CCL2 did not correlate with insulin sensitivity in this study, it is known that CCL2 does cause insulin resistance when mice are infused directly with this chemokine, and there is some evidence that this is also the case in adult humans although the evidence is not conclusive [23,25,26]. It is quite important to follow our patients to see if they develop insulin resistance and how CCL2 levels correlate with this. It remains to be seen if CCL2 can predict future metabolic outcomes including obesity, dyslipidemia and dysglycemia.

## Conclusions

In summary, this is a report of a previously unknown association of CCL2 as a potential biomarker of inflammation and fitness in overweight/obese children. Further characterization of its role in obesity-related comorbidities may open the door for more targeted interventions including more focused exercise programs, nutraceutical or pharmacological interventions that modulate CCL2 levels to tackle inflammation in obesity.

## Competing interest

The authors declare that they have no competing interests.

## Authors’ contributions

MCS & BWT conceived the idea and generated the hypothesis. JO and TN recruited participants and performed body measurements and filled questionnaires. JO performed the CCL2 ELISA. MCS, BWT and LT performed the statistical analysis, and MCS wrote the manuscript. MCS is the guarantor for the overall content of this manuscript. All authors revised earlier versions and approved the final version of the manuscript.

## Pre-publication history

The pre-publication history for this paper can be accessed here:

http://www.biomedcentral.com/1471-2431/13/47/prepub
